# Eagle Syndrome: A Rare Case of Atraumatic, Painful Cervical Neck Swelling

**DOI:** 10.5811/cpcem.2020.3.46284

**Published:** 2020-04-23

**Authors:** Cameron P. Worden, Sanjeeb S. Bhandari, Benjamin B. Cable, Damon R. Kuehl

**Affiliations:** * Virginia Tech Carilion School of Medicine, Roanoke, Virginia; † Virginia Tech Carilion, Department of Emergency Medicine, Roanoke, Virginia; ‡ Virginia Tech Carilion Clinic School of Medicine, Division of Otolaryngology, Department of Surgery, Roanoke, Virginia

**Keywords:** Eagle syndrome, atraumatic, fracture, airway impingement, hematoma

## Abstract

**Introduction:**

Painful neck swelling is a common emergency complaint but can present diagnostic challenges. Eagle syndrome is a rare clinical entity in which a pathologically elongated styloid process or ossified stylohyoid ligament produces a constellation of symptoms in the head and neck region.

**Case Report:**

We present the case of a 50-year-old male with a spontaneous, atraumatic fracture of an elongated styloid process associated with hematoma formation and radiological findings of airway impingement.

**Discussion:**

The classic triad for Eagle syndrome consists of unilateral cervicofacial pain, globus sensation, and dysphagia. Diagnosis of Eagle syndrome should be made based on a combination of physical examination and radiological findings. Treatment options vary based on severity of symptoms.

**Conclusion:**

Although more likely to be an indolent and progressive complaint, providers in the acute care setting should be familiar with Eagle syndrome due to the potential for a spontaneous fracture of an elongated styloid process to cause acute, painful neck swelling and life-threatening airway compromise.

## INTRODUCTION

Painful neck swelling is a common emergency complaint but can present diagnostic challenges. The complex anatomy of the neck makes isolating the cause of the swelling and pain a challenge for clinicians. History is critical in determination of the cause, especially the timing of onset of symptoms. While infection and trauma are common causes of painful cervical neck swelling, less common causes should also be considered in any differential diagnosis. Patients with acute, atraumatic pain and swelling of the cervical tissues (immediate to <24 hours) should also undergo investigation for pathology within other specific anatomic structures in this area of the neck including the following: sudden salivary gland obstruction; arterial rupture or dissection; thrombosis of deep (superior vena cava syndrome) or superficial veins (Lemierre’s syndrome); and possibly acute thyroid disease.^1^,^2^

When faced with this diagnostic dilemma, emergency physicians and specialists often turn to a computed tomography (CT) with intravenous (IV) contrast for assistance. While spontaneous injury to the cervical structures or ligamentous rupture or tear are rarely considered, and not routinely discussed in emergency medicine textbooks, fracture of the styloid process can occur spontaneously as part of a more complex constellation of symptoms known as Eagle syndrome, which leads to cervical neck pain and swelling.^1^ Here we present the first reported case of Eagle syndrome with a spontaneous, atraumatic fracture of an elongated styloid process resulting in hematoma formation and radiological findings of airway impingement.^3^

## CASE REPORT

A 50-year-old man presented to the emergency department (ED) complaining of progressively worsening swelling over the angle of his left jaw associated with difficulty speaking, described as hoarseness and pain with phonation, as well as difficulty swallowing. It began spontaneously one day previously, right after he felt a snap or pop. There were no triggering incidents, although he was unclear if he was talking or swallowing at the time. It was associated with a non-radiating, moderate intensity pain, just below the angle of the mandible on his left side that increased in severity when he moved his jaw to speak, turn, or swallow. Over the course of 12 hours he developed worsening anterior cervical neck swelling and progressive odynophagia, dysphagia, and hoarseness. His medical history was significant for hypertension and left-sided Bell’s palsy since 2014, which was associated with ipsilateral tinnitus.

**Image 1:**
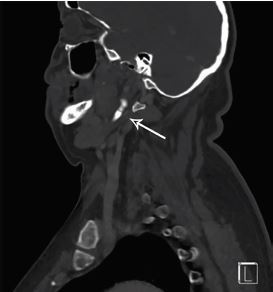
Computed tomography of the neck with intravenous contrast in sagittal view showing an elongated left styloid process with associated atraumatic fracture, indicated by arrow.

**Image 2:**
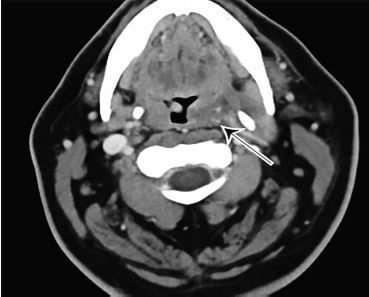
Computed tomography of the neck with intravenous contrast in axial view showing fluid tracking along the fractured left styloid process producing a mass effect on the left lateral hypopharyngeal wall, indicated by arrow.

On examination, he was hypertensive with a blood pressure of 187/105 millimeters of mercury, heart rate 91 beats per minute, temperature 98.4° Fahrenheit, and oxygen saturation 100% on room air. The patient’s physical exam was unremarkable with the exception of palpable and visible swelling to the soft tissues below the left side of the mandible and clear discomfort on swallowing. Concern for a vascular abnormality such as aneurysm or thrombosis prompted immediate CT of the neck with IV contrast. This imaging revealed a fracture though an elongated, calcified left styloid process (Image 1) with hematoma formation causing mass effect on the left lateral hypopharyngeal wall (Image 2).

**CPC-EM Capsule**

What do we already know about this clinical entity?

Eagle syndrome is a rare clinical entity in which a pathologically elongated styloid process or ossified stylohyoid ligament produces a constellation of symptoms in the head and neck region.

What makes this presentation of disease reportable?

This is the first reported case of Eagle syndrome with a spontaneous, atraumatic fracture of an elongated styloid process resulting in hematoma formation and airway impingement.

What is the major learning point?

There is a potential for Eagle syndrome to present as a spontaneous, atraumatic fracture of an elongated styloid process leading to acute neck swelling and life-threatening airway compromise.

How might this improve emergency medicine practice?

This report highlights an important differential in the workup of painful neck swelling that has the potential to lead to life-threatening complications if not promptly recognized.

The patient was preliminarily diagnosed with a spontaneous fracture of an elongated calcified styloid ligament with hematoma formation and was admitted to observation with otolaryngology (ENT) consultation for airway monitoring and the potential for surgical intervention if worsening airway impingement. He did well with no further progression of symptoms and was discharged after 24 hours. At follow-up with ENT services one week post-ED visit, swelling was markedly reduced and his pain had fully resolved. The spontaneous fracture was thought to be corrective and no further surgical intervention was required.^4^,^5^ He has had no further symptoms one year from his injury.

## DISCUSSION

The styloid process is a slender bony extension of the temporal bone projecting immediately anterior to the stylomastoid foramen. In a normal adult, the styloid process is approximately 2.5 cm in length, whereas an elongated styloid process is defined as > 3 cm.^6^ While it is estimated that approximately 4–28% of the general population have an elongated styloid process, few are symptomatic.^7^ Reasons for the elongation are poorly understood. Possible pathophysiological mechanisms include the following: 1) a congenital elongation of the styloid process due to the persistence of a cartilaginous element connecting it to the temporal bone; 2) ossification of the stylohyoid ligament; and 3) growth of osseous tissue at the insertion of the styloid ligament.^4^

Eagle syndrome is an uncommon and often-confusing clinical entity, likely due to the variable constellation of symptoms that can develop.^8^ It is characterized by a pathologically elongated styloid process or ossified stylohyoid ligament producing symptoms in the head and neck region. As first described by Watt W. Eagle in the 1930s, Eagle syndrome classically presents as a triad of globus sensation, dysphagia, and unilateral cervicofacial pain typically occurring after tonsillectomy.^6^ He proposed that scar tissue that developed as a consequence of the surgery around the mineralized complex resulted in compression of surrounding cranial nerves V, VII, IX, and X leading to chronically progressive symptoms.^6^ However, the current definition of Eagle syndrome has evolved to include a myriad of additional symptoms including carotid compression resulting in syncope and transient ischemic attacks, otalgia, tinnitus, odynophagia, and generalized cervicofacial pain that all derive from a pathologically elongated styloid process or calcified stylohyoid ligament.^8^,^9^,^10^

In the acute care setting, painful cervical neck swelling should include a differential of infection or trauma, as well as less common etiologies including sudden salivary gland obstruction, arterial rupture or dissection, thrombosis (Lemierre’s syndrome), acute thyroid disease, and ligamentous injury.^1^,^2^ In our patient, accompanying symptoms of dysphonia and dysphagia likely resulted from the compression of the left lateral hypopharyngeal wall and associated neurovasculature due to the hemorrhage and swelling in the parapharyngeal space from the styloid process fracture. Our patient did extremely well, but expanding hematomas, especially in the anticoagulated patient, make this uncommon and benign condition potentially life threatening in the acute phase of injury.

Diagnosis of Eagle syndrome should be made based on a combination of physical examination and radiological findings. The imaging method of choice is CT of the neck with IV contrast. Panoramic radiography may be diagnostic, although it does not narrow the differential in the acute setting.^11^ Treatment options for Eagle syndrome vary based on severity of symptoms. Non-surgical interventions ranging from simple reassurance to local corticosteroid injections can be successful for mild to moderate symptoms. For severe cases, surgical excision of the elongated styloid process or ossified stylohyoid ligament is recommended via a transcervical (extraoral) approach for proper visualization of anatomical structures and a decreased incidence of deep cervical space infection.^4^,^5^

## CONCLUSION

Spontaneous fracture or ligamentous injury of the styloid process should be considered in a patient presenting with acute onset, atraumatic, painful cervical neck swelling. The classic triad for Eagle syndrome consists of unilateral cervicofacial pain, globus sensation, and dysphagia. Although more likely to be an indolent and progressive complaint, providers in the acute care setting should be familiar with Eagle syndrome due to the potential for a spontaneous fracture of an elongated styloid process to cause acute painful neck swelling and life-threatening airway compromise. While history and physical exam findings are critical in suspecting the condition, CT imaging is required to confirm the diagnosis, exclude alternate serious clinical entities, and guide management.
